# Gender-Affirming Surgery as a Treatment Option for Gender Dysphoria in Adults: A Systematic Review of Ethical Reasons

**DOI:** 10.3390/healthcare14162605

**Published:** 2026-08-19

**Authors:** Bijosh Moolekkudiyil Baby, Yves De Maeseneer, Kris Dierickx

**Affiliations:** 1 Theological and Comparative Ethics, Faculty of Theology and Religious Studies, KU Leuven, Sint-Michielsstraat 4, Box 3101, 3000 Leuven, Belgium; yves.demaeseneer@kuleuven.be; 2 Centre for Biomedical Ethics and Law, Faculty of Medicine, KU Leuven, Kapucijnenvoer 7, Box 7001, 3000 Leuven, Belgium; kris.dierickx@kuleuven.be

**Keywords:** gender dysphoria, gender-affirming surgery, treatment, ethics, reasons

## Abstract

**Background**: Gender dysphoria denotes the psychological distress experienced by individuals due to the incongruence between their gender identity and their sex assigned at birth. Gender-affirming surgery (GAS) is one of the treatment options available for people with gender dysphoria. This study aims to identify and analyze all ethical reasons both supporting and opposing GAS as a treatment option for gender dysphoria in adults. **Methodology**: A systematic review of reasons was conducted in accordance with the methodology proposed by Strech and Sofaer (2012) and the PRISMA guidelines. The literature search was conducted in seven major online databases—PubMed, Scopus, Embase, Web of Science, Philosopher’s Index, ATLA Religion, and Index Theologicus—and in the Google Scholar search engine. **Results**: Twenty-nine articles published between 2003 and 2024 met the inclusion criteria. The ethical reasons identified in the included literature were grouped into five thematic categories: (1) the origin and nature of gender dysphoria, (2) reasons related to GAS as a treatment for gender dysphoria, (3) anthropological reasons, (4) reasons related to the effects of GAS, and (5) reasons stemming from traditional ethical principles. **Discussion**: The wide range of ethical reasons both supporting and opposing GAS as a treatment option for gender dysphoria in adults highlights the complexity of ethical deliberation in this area. This review provides a structured overview of the ethical reasons discussed in the literature, identifies important gaps, and suggests directions for future research.

## 1. Introduction

Gender dysphoria is defined in the latest version of the *Diagnostic and Statistical Manual of Mental Disorders (DSM-5-TR)* as “the distress that may accompany the incongruence between one’s experienced or expressed gender and one’s assigned gender” [1]. This distress is so intense that people with gender dysphoria earnestly desire and try to change their physical sex characteristics to match their gender identity, even to the point of considering self-mutilation or suicide as an escape [2,3,4,5]. According to the *DSM-5-TR*, the prevalence of a gender dysphoria diagnosis in the population based on treatment-seeking data is less than 0.1 percent for both males at birth and females at birth [1]. A range of social, psychological, behavioral or medical interventions (including hormone therapy and gender-affirming surgery) that are part of gender-affirming health care in general is designed to support people with gender incongruence to affirm their gender identity [6]. Among these, gender-affirming surgery has gained significant prominence in recent decades among people with gender dysphoria, the general public, and within the medical community and institutions [7].

Currently, the term gender-affirming surgery (hereafter, GAS) serves as a hypernym for “all surgical procedures that a patient wishes to receive to resemble the appearance of the opposite gender” [7]. Various terms such as gender affirmation surgery, gender reassignment surgery, sex reassignment surgery, sex change surgery, transgender surgery, genital reconstruction surgery, etc., are used synonymously with gender-affirming surgery in medical and ethical literature. We acknowledge that these terms reflect different conceptual and normative perspectives. Disregarding the nuances of these terms, we adopt ‘gender-affirming surgery’ (GAS) that is preferred in contemporary scientific literature. At the same time, to ensure methodological rigor, all relevant terminologies are incorporated into the search strategies employed across the various databases, thereby maximizing the identification of all articles pertinent to this review. It should be noted that not all individuals with gender dysphoria require, desire, or seek GAS as part of their treatment [8]. According to some available data, the prevalence of GAS among the transgender population ranges from 25% to 35% [9,10,11,12]. At the same time, these data are based on documented cases of GAS, and these figures may not reflect the actual data, as many cases of GAS have not been properly reported.

At present, GAS procedures and techniques are highly developed, widely practiced [11,13,14], achieve aesthetically satisfactory results, and continue to evolve along new avenues [7]. To our knowledge, no published literature has systematically examined the ethical arguments concerning GAS. (A recently published article addresses a related topic [15], exploring a similar but broader area of research; our study, however, differs significantly in terms of research question, scope, focus, methodology, literature selection and analytical approach.) Therefore, we undertake a review of reasons to identify and analyze all ethical reasons and arguments presented in the normative ethical literature regarding GAS as a treatment option for gender dysphoria in adults.

By synthesizing dispersed normative arguments, this review provides a structured overview of the ethical landscape surrounding GAS and contributes to a more comprehensive understanding of the ethical considerations relevant to its use as a treatment option for gender dysphoria in adults. The findings may inform clinical, ethical, and policy discussions by highlighting the range of normative reasons both in support of and in opposition to GAS. Furthermore, this review identifies areas of ambiguity within the existing literature and underscores the need for further normative and empirical research to facilitate more informed ethical deliberations on the topic.

## 2. Methodology

In this systematic review, we follow the methodology proposed by Strech and Sofaer [16] for the review of reasons. This involves running a systematic search for the literature on relevant databases, identifying and screening relevant articles based on predefined inclusion and exclusion criteria, and including articles that meet the required quality criteria for further analysis. This review is conducted and reported in accordance with the Preferred Reporting Items for Systematic Reviews and Meta-Analyses (PRISMA) guidelines [17] for systematic reviews. As a thorough check conducted prior to initiating our study indicated that no systematic review existed on the same topic, our study was not preregistered. However, to enhance the transparency, a post hoc registration documenting the title, methodology, and analytical procedures was deposited on the Open Science Framework (OSF) [18].

### 2.1. Review Question

This study addresses the following review question: Which ethical reasons have been given in the normative ethical literature for the view that gender-affirming surgery is or is not an ethically justified treatment option for gender dysphoria in adults?

### 2.2. Search Strategy

**Figure 1:**
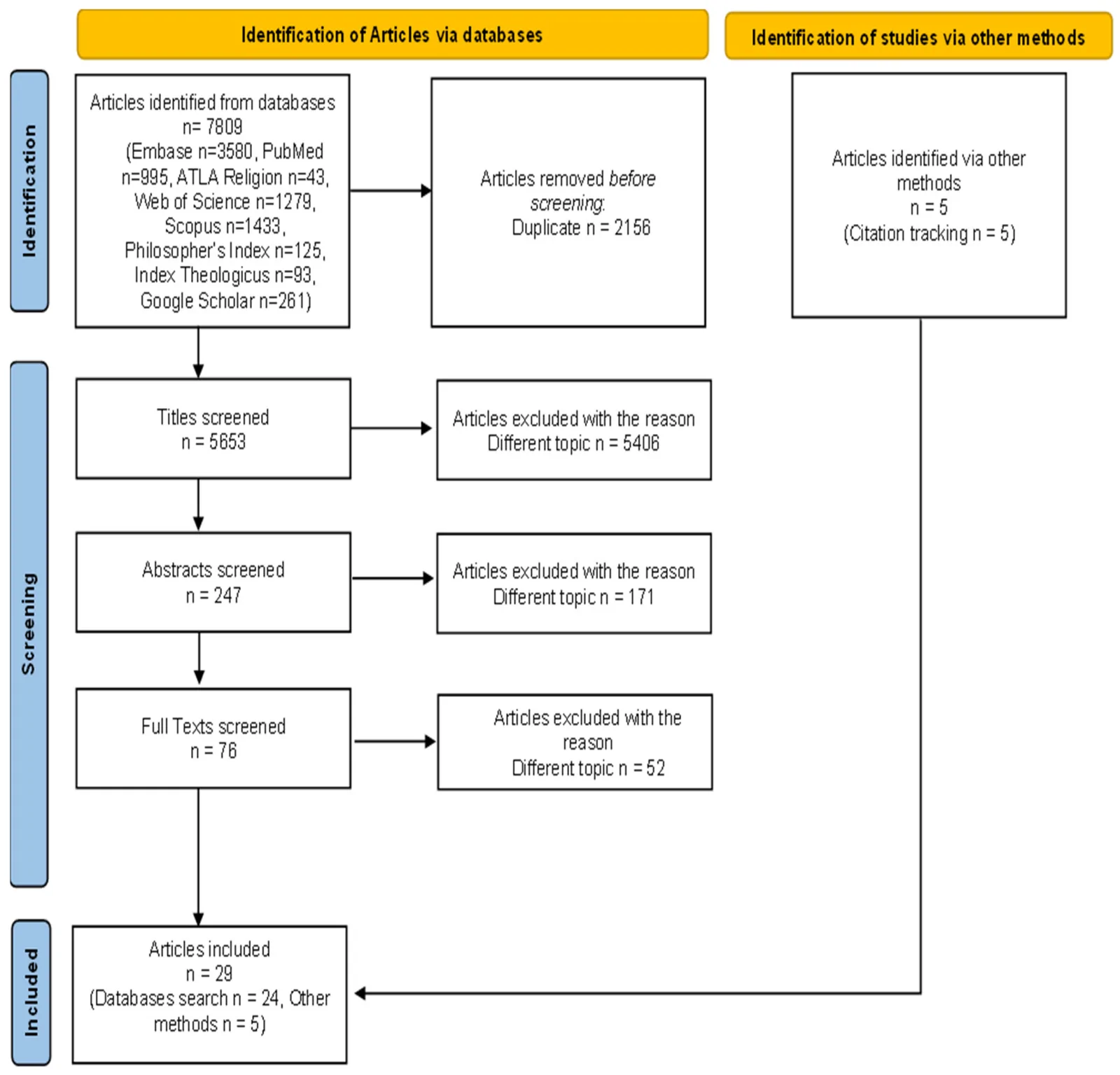
PRISMA flow diagram showing selection of literature for the review.

A literature search was conducted in seven major academic databases known to include ethical literature, namely, PubMed, Scopus, Embase, Web of Science, Philosopher’s Index, ATLA Religion, and Index Theologicus. The search string was developed using Boolean search operators (AND, OR, and NOT) on three groups of concepts, namely, Group A: Gender-affirming, Group B: Surgery, and Group C: Ethics. A primary string was developed for PubMed and then adapted for use in other databases. Search filters for language (English only), publication year (from 2003 to 2022) and species (human only) were applied, if possible, to reach a manageable search result. Table S2 of the Supplementary Materials presents the concepts and associated terms used to develop the literature search strategy, and Section S1 provides the search strings employed across the various databases. A supplementary search was conducted in the Google Scholar search engine to ensure that no relevant literature was overlooked. Given the search engine’s limitation in supporting complex search strings, multiple combinations of keywords representing the core concepts of the review were used. For each search, the first 25 pages of 20 results per page were screened, and potentially relevant titles were saved to the Google Scholar library. These records were incorporated into the study selection process. In addition to this primary database and Google Scholar search, citation tracking was also performed to identify additional potential studies. The search results were exported and compiled using EndNote Reference Manager (version 20) software, and the duplicates were removed according to Falconer’s instructions [19]. Applying the predetermined inclusion and exclusion criteria, title, abstract, and full-text screening were subsequently performed on the remaining publications. The search was conducted between 13 February 2023, and 21 March 2023. Due to the extended study and publication timeline, prior to finalizing the manuscript, a subsequent literature search was performed on 15 December 2025, using the same search strategy to identify potentially eligible studies published during the intervening period. This second search was restricted to studies published between 2023 and 2024. The search strings were developed by B.M.B. in consultation with the other researchers. B.M.B. further conducted the database search, title screening, and abstract screening, while the entire process was reviewed by the research team. The inclusion of the articles was finalized through discussion among all researchers, where discrepancies were resolved by consensus. Figure 1 shows the PRISMA flow diagram for the initial literature search process for this review. The subsequent literature search between 2023 and 2024 identified no additional eligible studies and is therefore not included in this diagram.

### 2.3. Inclusion and Exclusion Criteria

An article is considered for inclusion only if it strictly adheres to the following inclusion criteria:

1. Literature concerning people with gender dysphoria;
2. Literature concerning adult people;
3. Literature concerning gender-affirming surgery;
4. Literature including ethical discussions on gender-affirming surgery;
5. Literature with normative/theoretical elaborations;
6. Literature published in English;
7. Literature published between 2003 and 2022 (For the subsequent search, the literature published between 2023 and 2024).

Criterion 1 excludes the literature on individuals undergoing similar reconstructive surgeries to treat disorders of sexual development (DSD), cancer, post-accident injuries, or for cosmetic reasons from this review. However, some literature is included if it contains a good discussion of gender dysphoria in comparison to the above-mentioned clinical conditions. Criterion 2 excludes the publications on gender dysphoria that only discuss issues relating to children, adolescents, and minors. Criterion 3 excludes ethical discussions on gender-affirming health care in general, as well as discussions of other specific management options for gender dysphoria, such as cross-dressing, behavioral therapy, hormone therapy, and psychotherapy, which are part of gender-affirming health care, along with GAS [6]. While including the literature containing explicit ethical arguments, moral reasons, or elaborations of ethical principles relevant to the research question, criterion 4 excludes the literature that contains only medical or technological details of the surgery. It also excludes the literature focusing upon socio-cultural, political, phenomenological, and legal discussions on gender transition. Focusing on normative (argument-based or reason-based) ethical literature, criterion 5 excludes the purely empirical literature on GAS. By normative ethical literature—also referred to as argument-based or reason-based literature—we mean theoretical “literature that includes normative and ethical considerations, such as ethical arguments, concepts, principles, or recommendations” [20]. This stands in contrast to empirical literature that reports empirical evidence obtained through qualitative or quantitative research methodologies to inform ethical conclusions.

### 2.4. Data Extraction and Analysis

We used NVivo software (Version 1.7.1) to support the data extraction, coding, and synthesis of the included articles. While these activities were conducted by the researchers themselves, the software served as a tool to host the data sources, organize codes systematically, and facilitate the analytical process by providing easy access to various codes. Using an adapted version of abductive thematic analysis by Thompson [21], we carried out the following steps: familiarization with the literature, extraction of codes, preparation of the codebook, development of higher codes, and identification of themes for data synthesis. Familiarity with the literature was achieved through repeated reading of the texts. A predefined data extraction criterion was used, whereby all text passages containing ethical reasons related to the acceptability or unacceptability of GAS as a treatment option for gender dysphoria were extracted verbatim from each included article. Each extracted passage that contained an ethical reason was referred to as a “reason-mention” according to the schema of Strech and Sofaer [16]. These reason-mentions were assigned descriptive codes inductively reflecting their content. The resulting codes were subsequently refined through comparison and consolidation with similar codes grouped under a common code. These refined codes corresponded to the “narrow reasons” in the Strech and Sofaer schema. A codebook was created to provide clarity and structure for these assigned codes for further analysis. The codes (narrow reasons) were then grouped into higher codes corresponding to the “broad reasons” and “reason types” of the Strech and Sofaer scheme. Overarching themes (“categories,” according to the Strech and Sofaer schema) were further identified from these broad reasons to synthesize the findings. The complete codebook, comprising the narrow reasons, broad reasons, and categories, is available in Table S1 of the Supplementary Materials. As this review focused on peer-reviewed academic journal articles published in major academic databases, all articles identified through the database searches that met the inclusion and exclusion criteria were included without any further critical appraisal of their study quality. Primary coding was performed by B.M.B. This was independently reviewed on the primary data by Y.D.M. and K.D. Subsequently, all researchers convened to identify discrepancies. After listening to each researcher’s reasoning, further discussions were held to reach a consensus. Once consensus was achieved, the discrepancy was considered resolved.

## 3. Results

Twenty-nine articles were selected in the literature search and reviewed for ethical reasons related to GAS as a clinical management option for gender dysphoria. The details of the included articles are given in Table 1.

**Figure 2:**
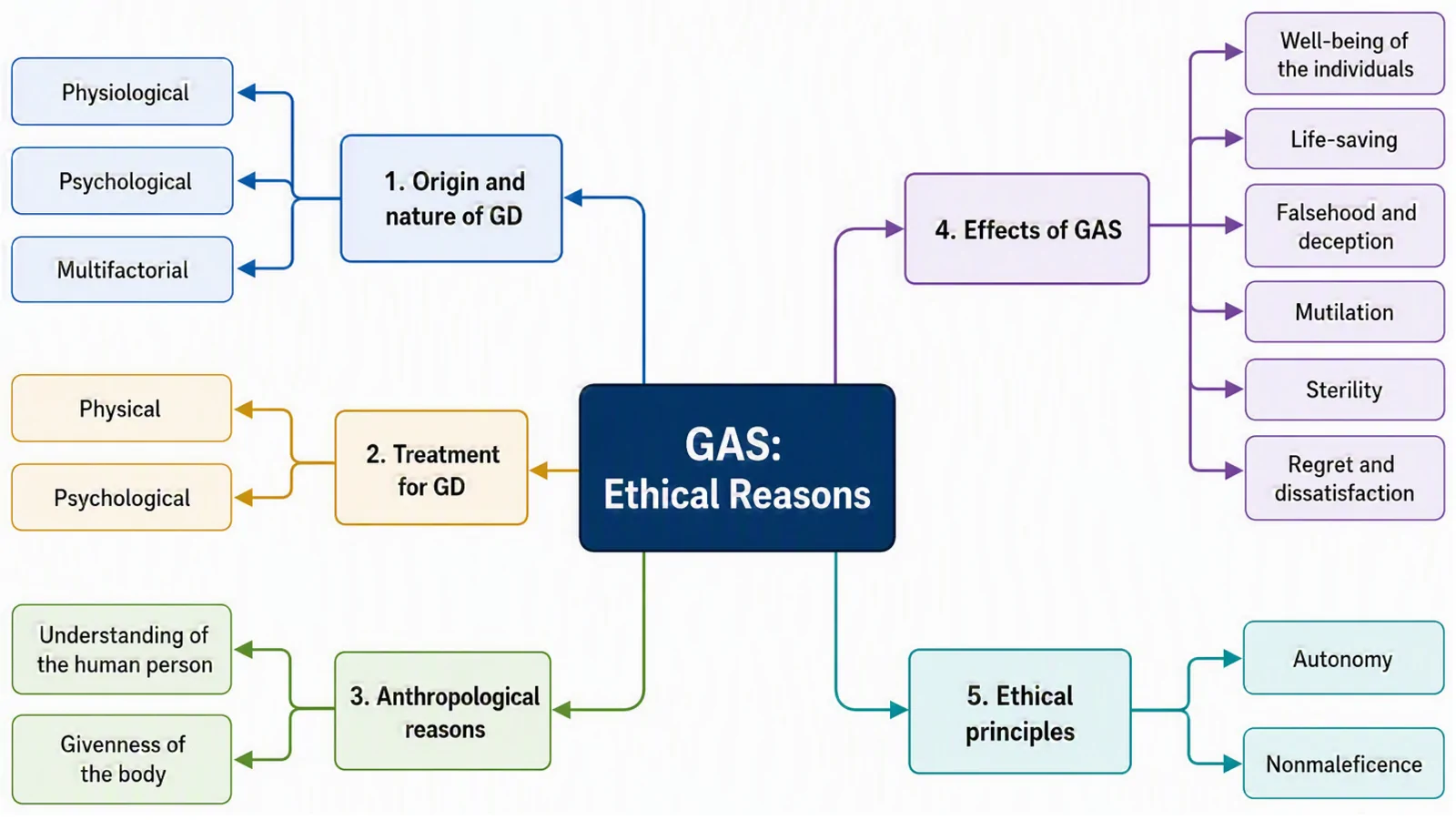
Five categories and their corresponding broad reasons identified in the given literature.

Following Strech and Sofaer’s methodology [16], this review examines all ethical reasons, both for and against GAS as a treatment option for gender dysphoria in adults, as found in the included articles, regardless of the personal opinion of the author(s) or the overall argument of the article. This means that the references in the brackets following a direct quote or a paraphrased text indicate only that the articles discussed a specific reason; they do not represent the overall position of the author(s) or the articles. However, the opinion of the author(s) regarding the acceptability of GAS is shown in Table 1. Similarly, in accordance with this methodological approach, all ethical reasons identified in the literature were included irrespective of their origin, quality, scientific basis, or the ethical weight of the underlying argument. At the same time, it is important to bear in mind that, as shown in Table 1, a substantial portion of the included articles is drawn from theological and philosophical ethical literature. A total of 231 reason-mentions (excerpts containing a reason) is identified in this review, covering eighty-four narrow reasons in relation to GAS, which are grouped into fifteen broad reasons. They are further grouped into five major categories, namely, the origin and nature of gender dysphoria, reasons related to GAS as a treatment for gender dysphoria, anthropological reasons, reasons related to the effects of GAS, and reasons stemming from traditional ethical principles. Figure 2 shows the broad reasons and their corresponding categories. These categories are not mutually exclusive, and some overlap exists among categories, as ethical reasons frequently rely on the same underlying assumptions. Such overlap reflects the multidimensional nature of normative arguments. Moreover, the frequency of reasons does not indicate the ethical or scientific validity of the underlying arguments. The five categories are discussed in detail in the following sections.

### 3.1. Origin and Nature of Gender Dysphoria

The first category of reasons is related to the origin and nature of gender dysphoria whether it is physiological, psychological or multifactorial. A total of twenty-eight reason-mentions were found in eleven articles, which are summarized below under three broad reasons.

#### 3.1.1. Physiological Origin of Gender Dysphoria

Some of the reviewed articles include reasons that suggest a physiological—physical or biological, as synonyms in the literature—origin of gender dysphoria. One reason found in the literature says that the primary cause of distress in people with gender dysphoria is the presence of physical sex organs that they find incompatible with their inner gender identity [4,27,41]. Some reason-mentions note that this distress is characterized as a strong feeling of “being trapped in the ‘wrong’ body” [41,47]. Some reason-mentions further appeal to a physiological origin, suggesting differences in brain structures, variations in genes [29], or neurodevelopmental factors such as a sex-atypical brain: the sex organs being not typical to the brain sex [23]. Consequently, surgery is not the construction of a new sex but the uncovering of the hidden true sex [27]. However, a counter reason found in the literature says that basing gender dysphoria solely on biological factors leads to biological determinism—the idea that biology decides one’s destiny, and the person has no power of self-determination [38,39].

#### 3.1.2. Psychological Origin of Gender Dysphoria

In contrast to the physiological origin view, some literature reports a reason that there is no obvious and empirically well-established biological cause for gender dysphoria, and for this very reason, it is presumed that gender dysphoria has a psychological origin instead [4,27,29,33,41,42,49]. Rejecting the ‘trapped in a wrong body’ narrative, it is said: “There is nothing wrong with the body. The problem lies in the mind’s perception and acceptance of the body. The mind is what is in need of intervention, not the body.” [39].

#### 3.1.3. Multifactorial

Some reason-mentions in the reviewed literature hold that gender dysphoria is multifactorial: psychosexual, genital, hormonal, developmental, social, and environmental [4,39,44]. Reason-mentions found in the literature suggesting a multifactorial origin remark that the physiological and psychological elements are interrelated in the development of gender dysphoria [4,49]. To those who insist on the physiological origin of gender dysphoria, this group of reasons says that physiological causes are probably a small factor among many others [38,39]. Given the multifactorial origin, one reason-mention says that the origin or the basis of gender dysphoria is not a good argument in the debate on the justification of GAS [39].

### 3.2. Reasons Related to Treatment for Gender Dysphoria

The discussion of whether the origin of gender dysphoria is physiological, psychological or multifactorial leads to the question about the appropriate treatment. Accordingly, the literature reports two main options: physical interventions, including GAS, and psychological treatments such as psychotherapy. A total of fifty-nine reason-mentions were found in twenty articles, presented below under two broad reasons.

#### 3.2.1. Physical Treatment

Reason-mentions that consider gender dysphoria to have a physical basis, specifically caused by the ‘wrong’ sex organs, suggest that “surgery is a morally permissible treatment for alleviating the distress of being trapped in the ‘wrong’ body” [41] by correcting those ‘wrong’ organs, which psychological intervention cannot achieve [4,29,41,46]. From this perspective, GAS is considered either to provide the missing organs or to remove the organs that the person accidentally possessed [22], thereby curing the discordance between the brain sex and the genital sex [23].

However, reason-mentions that view gender dysphoria as a psychological issue do not consider GAS to be an appropriate treatment option for gender dysphoria. Many reason-mentions are found in the literature in relation to this view. Some reason-mentions say that the surgery does not really change a person’s sex [32]. It is impossible to change a person’s karyotype and the effects of prenatal hormones on the brain through surgeries on the genitals [26,33,44,49]. Another set of reason-mentions notes that the sexual identity revealed in the sex organs is essential to the human person, and simply altering the sex organs does not change the underlying reality [42,46]. Some other reason-mentions in the same vein point out that even though the surgery allows individuals to simulate sexual intercourse, the organs created in the GAS are only imitations and are not functional for the reproductive aspects of human sexuality [4,23,27,31,33,45,46,48,49]. As it does not lead to entering into real sexual unions and begetting offspring, GAS is contrary to the institution of marriage [4,29,31,38,46]. According to this group of reason-mentions, “the trapped in the wrong body narrative” stems from a disordered perception of the self, and the surgery involves collaboration with a mental disorder [30,32,33,45]. Further, one reason-mention in this line argues that there is no convincing empirical evidence for the existence of a sex-atypical brain [47]. Some other reason-mentions say that GAS thwarts the possibility of self-acceptance and natural healing, as well as preventing the development of other therapies, particularly psychotherapies [33,42,46].

#### 3.2.2. Psychological Treatment

Many reasons that oppose physical treatment implicitly support psychological treatment. Some reason-mentions explicitly state that gender dysphoria needs to be corrected by psychotherapy [27,41,42,44]. Many reasons are listed in the literature specifically for promoting a psychotherapy approach over surgery. First, psychotherapy is in accordance with the view that physiology is an immutable trait. Second, psychotherapy avoids the medical risks associated with surgery. Third, many people with gender dysphoria, especially children, eventually become comfortable with their birth sex. Fourth, psychotherapy respects the ethical obligation to ‘do no harm’ by allowing individuals to continue in their natal sex. Fifth, psychotherapy avoids the ethical risks associated with obtaining informed consent for surgery from a psychologically impaired person [44]. One reason-mention acknowledges the current limitations of psychotherapy in treating gender dysphoria while remaining optimistic about the possibility of discovering an appropriate and effective psychological solution in the future [42].

### 3.3. Anthropological Reasons

In discussing the ethics of GAS as a treatment option for gender dysphoria, some of the literature includes reasons related to anthropological questions about the human person. This category analyzes two broad reasons found in the reviewed articles, namely, reasons related to the understanding of the human person and the givenness of the body. There were twenty-five reason-mentions found in fifteen articles in this category.

#### 3.3.1. Understanding of the Human Person

Related to the reasons cited under the category of origin and nature of gender dysphoria, some of the literature discusses an anthropological question that has ethical implications, framed as the dispute between a dualistic and unitive view of the human person. Based on broader philosophical and theological perspectives on the human person as having a corporeal and a spiritual component, a dualistic understanding assumes a sharp distinction between body and soul—self, mind or psyche are used as synonyms in the literature reviewed. (The concepts are discussed here as they are found in the reviewed literature. Their use reflects the anthropological frameworks adopted by the respective authors.) According to this view, body and soul can be sexed independently, and they can be misaligned in such a way that a female soul is found in a male body or vice versa [34,38]. Furthermore, a person’s true identity lies in the soul and not in the body [29], and it is the body that needs to be changed according to the soul if there is a misalignment [40]. Against this dualistic view, a unitive understanding of the human person, which sees the soul as the blueprint or vivifying principle of the actually existing sexed body, maintains that the body and the soul together form a composite unit that cannot be separated from each other [23,26].

With regard to the body and soul in the human person, reason-mentions in support of GAS maintain the view that “the ‘real’ self is not the body as given but merely the ‘self’ as perceived” [23]. Conversely, the reason-mentions that reject GAS, adhering to a unitive understanding of the human person, allege that the proponents of GAS maintain a body–soul dualism—an idea that the former regard as incompatible with the constitution of the human person [4,26,29,38,40,42]. According to this group of reasons, the dualistic view contradicts “objective truths about the nature of the human person” [40], and implies an ontological disintegrity in the human person [23,38], because the spiritual soul is considered the essential form of the human body [35,36,39,43].

On the other hand, one reason-mention in this line says that the human person must be able to enjoy the unity of body and soul when gender dysphoria causes a disintegrity. At this point, GAS is considered to be “a story of integration” that attempts to restore the unity of body and soul [38,46].

In addition to these arguments about the relationship between body and soul, some reason-mentions consider the relationship between sex and gender. Reason-mentions that regard biological sex as the real sex consider the sex–gender dualism to be impossible, since there is no objective reality of gender in the absence of sex organs [26,35,47].

#### 3.3.2. Givenness of the Body

The reviewed literature includes another anthropological reason, namely, the givenness of the physical body and natal sex in the ethical debate concerning GAS. A group of reason-mentions suggests that GAS not only fails to accept the goodness of the given body and sex [29] and the given personhood manifested in one’s sexuality [4,48], but also involves the devaluation and dishonoring of the given body [30,38]. One reason-mention notes that by rejecting the given body, sex, and the gender associated with birth sex, and choosing their own gender, GAS subjects depend on the absolutization of their own consciousness and act on their own subjective feelings [38]. Similarly, according to another reason-mention, GAS entails an erroneous anthropology expressed through the belief that a person’s sex can be changed [4].

In contrast, some supporters of GAS also uphold the value of givenness in a different way. “But for them it is the gender that is ‘given’ and therefore should not be changed, rather than bodily sex.” [29].

### 3.4. Reasons Related to the Effects of GAS

The reviewed literature points out positive and negative effects and consequences of the surgery when debating the ethical acceptability of GAS as a treatment option for gender dysphoria. Some of the effects of the surgery have already been mentioned in the section on physical treatment. The reasons in this category examine only the effects and consequences of GAS that are not mentioned in other sections. A total of ninety-three reason-mentions were found in twenty-seven articles, presented below under six broad reasons.

#### 3.4.1. Well-Being of the Subjects

Some reason-mentions found in the literature hold the view that GAS is a beneficial medical intervention for people experiencing gender dysphoria. A few reason-mentions state that by alleviating the intense distress associated with gender dysphoria, GAS can greatly improve the individual’s psychological well-being [4,33,39] and “should be considered as having positive moral weight when one is evaluating the moral permissibility” [41]. Another reason-mention notes that in demanding GAS, individuals with gender dysphoria are expressing their desire to ‘look normal’ and ‘enhance beauty’ just like other people, with the only difference being that their requests are ‘cross-gendered’. The surgery helps to enhance their self-image and self-esteem [43].

At the same time, some reason-mentions deny that GAS is a beneficial treatment for gender dysphoria based on the reasons already mentioned in previous sections. They specifically highlight the inadequate data proving the long-term effects of the surgery [4,23,26,27,31,33,37,45,47,49], the inability of GAS to remove the underlying psychological issues [23,27,38,44,45,47], and the inability to create functional organs [4,33].

#### 3.4.2. Life-Saving

It is generally assumed that at the verge of intense distress from cross-gender identification, suicidal ideation and a tendency toward self-mutilation are part of gender dysphoria [4,33,45]. Given the high rate of suicide among transgender persons, which can reach 50 percent higher than that in the general population, some reason-mentions consider GAS to be a life-saving treatment [49]. According to this reason, in the absence of a promising alternative treatment option for gender dysphoria, GAS can be considered a last resort for people with this condition to reduce their distress and to create happiness [4,26]. Furthermore, one reason-mention states that GAS can be permitted as an exception, as clinical conditions generally allow for exceptions when other treatment options have failed [4].

On the other hand, the reason-mentions that oppose the surgery state that the post-surgery suicide rate is also high, nearly 20 percent higher than that of the general population which in essence questions the long-term efficacy of GAS [26,46]. Some other reason-mentions consider the suicidal ideation and the tendency toward self-mutilation to be part of a mental disorder [4,33,45]. Another reason-mention points out that individuals with gender dysphoria sometimes employ a suicide threat as blackmail to qualify themselves for GAS [39].

#### 3.4.3. Falsehood and Deception

In line with the argued impossibility of sex change, another set of reasons is found in the literature suggesting that GAS involves falsehood and leads to deception. One reason-mention comments that people with gender dysphoria act on a self-perception of being a member of the other sex based on their subjective feelings [23]. Some reason-mentions say that the demand for GAS is based on a fantasy or a delusion that an individual can change their sex and obtain the sex organs of their desired gender through the surgery. Accordingly, the effect of the claimed sex change in GAS would only be the fulfillment of a fantasy desire to establish a false identity that is far from reality [4,33,39]. Within this context, GAS, instead of curing the underlying psychological issue, attempts to reduce psychological distress based on this self-deception [39,46]. Furthermore, some reason-mentions note that the falsehood created by the surgery deceives not only oneself but also others by presenting oneself as a member of the other sex [33,34,39]. It becomes a massive deception of others when someone who has undergone GAS marries someone of the same birth sex without informing the latter of the surgery [33]. One reason-mention observes that transwomen, who were not born female and never experienced growing up as women, are betraying women when they describe what it means to be a woman [33]. Reflecting further on the concept of deception, one reason-mention indicates that if deception in itself is bad, then any effort to adopt the appearance of the other sex, whether through mannerisms, behaviors, cross-dressing, surgery, or any other means is fundamentally bad [4]. Another reason-mention questions the credibility of the claims of the individuals seeking GAS, asking whether they truly believe they belong to the other sex or whether they say so, having learned that this is the only way to qualify themselves for the surgery [33].

On the other hand, there are also reason-mentions that suggest the people with gender dysphoria cannot be regarded as fantasy-driven or delusional. “Delusion is a fixed belief in something untrue”, but people with gender dysphoria only feel like a member of the other sex rather than truly believe it [49]. At the same time, some reason-mentions maintain that the aforementioned deception is not always necessarily false or wrong [26]. A well-understood legal fiction may not be deceptive, as in the case of legal adoption, where children who are not biological offspring are treated as true sons or daughters [4]. Another reason-mention in favor of the surgery proposes that the individuals who undergo GAS can be truthful even after the surgery, as in the case of a male-to-female person who rightly claims that she is not a ciswoman, but a transwoman [4]. In addition, a treating doctor can also uphold truthfulness when performing GAS if he or she does not share the individual’s self-deception but acts on the doctor’s own truthful reasoning [4,26].

#### 3.4.4. Mutilation

One of the evident effects of GAS that raises ethical concerns is that it involves the mutilation of functional reproductive organs of the body, since it violates the ethical duty to protect and preserve bodily and functional integrity. Some reason-mentions explicitly deny the ethical permissibility of GAS, arguing that it leads to the destruction of properly developed sex and reproductive organs along with the destruction of their normal functions [4,30,32,33,35,38,39,42,49]. Consequently, they contend that the mutilation involved in GAS results in “trading functioning organs for ‘organs’ that are largely nonfunctional” [41].

On the other hand, some reason-mentions bring forth the principle of totality—a traditional ethical principle based on the part–whole relationship in which the good of the part is subordinate to the good of the whole [50]—to justify the reconstruction of healthy sex organs for the good and well-being of the whole person [4,23,30,34]. One reason-mention notes that the part–whole relationship implies more than just the benefit of the body, and the human being as a whole includes the physical, psychological, social, and spiritual dimensions of a person [37].

However, there are also reason-mentions suggesting that the principle of totality cannot be properly applied in the case of GAS. Various reasons—some of which are already mentioned in previous sections—are found in the reviewed literature, such as: (1) The surgery is a direct attack on the physical integrity [4,37,39,45]. (2) There is no part–whole relationship in the case of gender dysphoria; empirical evidence does not support the long-term effects of GAS [4]. (3) There is no clear knowledge about the disjunction between mind and body, and the claimed cure achieved by conforming the body to the mind [25,40]. (4) GAS is based on the false claim that sex can be changed [26,48]. (5) GAS negates the goodness of the given personhood [48]. (6) It is not the healthy organs that cause distress and GAS does not treat the source of pathology [30]. (7) There are additional social and ethical challenges, such as the implications for marriage or living as a surgically disabled person [29]. (8) GAS does not respect the intrinsic teleology of sex organs [42].

#### 3.4.5. Sterility

In relation to the reconstruction of sex organs, reason-mentions opposing GAS point out that the surgery causes sterilization and leads to infertility [24,26,33,48]. One reason-mention indicates that by causing harm to the normal functions of reproductive organs, GAS compels the subjects to adopt artificial reproductive methods instead of natural reproduction [4].

However, there is a counter reason that GAS is not a direct sterilization because it is not directed (intended) toward sterilization, rather, it is intended only to reduce the distress caused by gender dysphoria [4]. Another reason found in the literature defends the ethical acceptability of GAS, especially genital surgeries which sterilize the person, on the basis of the principle of double effect—a traditional ethical principle that is used to analyze the justifiability of an action having two effects, one of which is foreseen but not intended, and for which there is a proportionate reason to permit it. The four conditions of the principle of double effect are explained in the reviewed literature as: “The action undertaken must be good or at least neutral; the desired effect must be good; the bad effect must not be the means to the good effect and the action undertaken must be proportionate to the desired good outcome” [22]. These conditions are applied here as follows: GAS is neutral. There are two effects of the surgery. The good effect is the achievement of the sex according to the wish of the individual and thereby the relief of mental distress. The bad effect, namely the sterilization, though foreseen, is not intended and is not a means to achieve the good effect. The bad effect is proportionate to the good effect [22].

Nevertheless, some other reason-mentions disagree with the justification of GAS through the application of the principle of double effect. They opine that the act, namely the surgery, is not ethically neutral [23,34,40]. The good effect, namely the alleviation of mental distress in the long term, is also under scrutiny [26,40]. Reiterating the arguments presented in the previous sections, some reason-mentions point out that applying the principle of double effect in the case of GAS presumes that gender dysphoria is a physical or biological disorder and reinforces body–soul dualism [34,40].

#### 3.4.6. Regret and Dissatisfaction

The irreversible nature of GAS attracts ethical discussion concerning regret after the surgery due to dissatisfaction with the surgical outcomes [24]. Dissatisfaction and regret may lead to the demand for reversal of the surgery [24,26,32,38].

At the same time, reason-mentions in support of GAS observe that the rate of regret following the surgery among GAS subjects is very low [37,38,43]. Furthermore, the dissatisfaction is not with the surgical outcomes but rather “of society’s inability to accept transsexual persons who have had the surgery and due to the fact that many transsexual persons are forced to hide their pasts and therefore live in constant fear of being found out” [38].

### 3.5. Reasons Related to Ethical Principles

When discussing the ethics of GAS in relation to the treatment of gender dysphoria, some reason-mentions explicitly state certain traditional philosophical, theological, and medical ethical principles by name. In this review, we identify certain reasons stemming from ethical principles such as the principle of totality, the principle of double effect, autonomy, beneficence, and nonmaleficence. Because the principles of totality, double effect, and beneficence have already been discussed in earlier sections, they are not repeated here. This category, therefore, focuses only on reasons grounded in autonomy and nonmaleficence. There were twenty-six reason-mentions in fourteen articles under this category.

#### 3.5.1. Autonomy

Autonomy, as a principle in medical ethics, ensures the self-determination of an individual with sufficient rationality and freedom to undergo any medical intervention. Autonomous persons are authorized to make proactive decisions about their own health care [41]. Based on this principle, some reason-mentions propose that GAS is ethically permissible, if the subjects are competent to make decisions and have the financial means to pay for the surgery [29,41].

On the other hand, some other reason-mentions strongly oppose the application of the autonomy principle in the case of GAS. For example, one reason-mention considering gender dysphoria as a mental disorder questions the competence of people with gender dysphoria to legitimately exercise their autonomy regarding the surgery [28]. In the same vein, some reason-mentions question the freedom of the individual. For them, the freedom of an individual is not absolute but must be in conformity with the objective truth [38,40]. One reason-mention notes that the autonomy of the individual is a sensitive area, “because sometimes the individual’s desires, hopes and expectations might not correlate with the reality” [24]. Further, one reason-mention opposing GAS observes that the presentation of GAS as a medical necessity also has an impact on autonomy, as it affects the volitional power of the will to refuse the surgery [38]. According to another reason-mention, the justification of GAS based solely on the autonomy principle emphasizes a want-based analysis, rather than a need-based analysis [44].

#### 3.5.2. Nonmaleficence

The principle of nonmaleficence proposes ‘first do no harm’. Pointing out the mutilation and destruction of healthy functional organs involved, some reason-mentions consider that GAS is a violation of the principle of nonmaleficence [22,29,41,42]. One reason-mention states that by overlooking the impossibility of sex change, GAS does more harm than good for the person [44]. Another reason-mention remarks that GAS results “in significant pain and suffering, incurs real, unjustifiable risks to patients, and does not address the real psychological problems” [33]. Based on the results of some studies, one reason-mention observes that “there is empirical evidence that the long-term effects of [GAS] are deleterious” and that the surgery increases mental difficulties [26]. According to some reason-mentions, the surgery involves physical comorbidities and side effects such as the risk of infection, excessive scarring, loss of function at the tissue donor site, loss of reproductive capacity, and an increased risk of stroke or heart attack, etc. [41,47]. Furthermore, presenting surgery as a solution to gender dysphoria prevents the individuals from revealing underlying problems that could be solved without the surgery [33].

## 4. Discussion

In our study of the large number of articles dealing with GAS in the searched databases, it is striking that the vast majority of the literature discusses the medical and technological aspects of GAS, with only a limited number of articles focusing on the ethical dimensions of the surgery. This review’s focus excludes the literature on gender-affirmative health care as a whole [51,52,53,54] and the literature that addresses broader health care delivery considerations related to gender-affirming care [55,56,57,58,59,60,61,62,63,64]. Among the available ethical literature related to GAS, many articles discuss the topic of surgery exclusively on children, adolescents, or youth. However, ethical questions surrounding GAS in minors differ substantially from those concerning adults, particularly regarding decision-making capacity, informed consent, parental involvement, and the evolving nature of identity development [62,65,66,67,68,69,70,71]. Similarly, there are also empirical studies that only implicitly contain ethical considerations. While some of them may contain ethical arguments equally relevant to GAS in adults, addressing them would require a separate analysis that is beyond the scope and feasibility of this review. Of the articles included, the majority are published from a theological perspective (seventeen articles). Given the rapid development of GAS technology and the increasing number of individuals opting for the surgery in recent years [13,72,73,74,75], the relatively limited amount of ethical literature on GAS from non-theological perspectives highlights a significant gap in the field. This scarcity underscores the need for more focus and reflection on the ethical dimensions of GAS as a treatment option for gender dysphoria in adults.

Further, the composition of the included articles warrants a cautious approach while interpreting the findings of this review. As shown in Table 1, a substantial portion of the included articles originates from theological and philosophical ethical perspectives. The review did not selectively include the Christian or theological literature. Rather, the review systematically searched the available normative literature meeting predefined inclusion criteria. Therefore, the composition of the included literature is itself a finding about the state of the field, and not the result of a methodological weakness. At the same time, this composition might have likely influenced both the nature of the ethical reasons identified and their relative frequency within the review. Consequently, the predominance of reasons reported in this review should not be interpreted as reflecting the overall balance of opinion within contemporary bioethical or broader clinical ethics. Rather, this review reflects the characteristics of the body of the published normative literature that met the inclusion criteria.

Similarly, the results of this review show that the majority of the articles were written by American (twenty-two articles), Australian (three articles), or European (two articles) authors, while only a few were written by African (one article) or Asian (one article) authors. Given that less than 0.1 percent of the population worldwide [1] experiences gender dysphoria, this observation shows an uneven representation of ethical perspectives on GAS as a treatment option for gender dysphoria across different regions of the world in scientific literature. This uneven geographical distribution warrants caution when interpreting the findings, as ethical deliberations are often shaped by cultural, religious, legal, and health care system contexts. Consequently, ethical reasons that are prominent in the American, European, or Australian literature may not fully capture the ethical perspectives from other world regions where the issues related to gender change are understood differently.

In the reviewed articles, none of the authors identify themselves as individuals with personal experience of gender dysphoria or GAS. Furthermore, of the reviewed authors, only Moschella [46,47] explicitly refers to the real-life experiences and voices of individuals with gender dysphoria. Consequently, the ethical reasons identified in this review may not fully represent the perspectives, priorities, or lived experiences of individuals with gender dysphoria themselves. This absence constitutes an important contextual consideration, particularly in light of the growing emphasis on participatory and patient-centered approaches in health care research. It therefore highlights a notable gap in the broader ethical debate on gender-affirming surgery. However, we do not further examine the implications of this absence, as the purpose of this review was to systematically identify and analyze ethical reasons presented in the scholarly literature rather than to investigate the representation of different stakeholder groups, including people with gender dysphoria. Future research may usefully explore how the inclusion of lived-experience perspectives could influence ethical discourse on gender-affirming surgery.

As a whole, this review aims to report on every reason and each individual stance on the topic found in the included literature while exploring the ethical complexities of the discussion for further ethical analysis. Observing that there is far from unanimity among the reviewed authors, it shows that of the eighty-four narrow reasons (231 reason-mentions) identified in this review, sixty are against GAS and twenty-four are in favor. Similarly, the majority of the reviewed articles (twenty-one in total) are against GAS. The numerical predominance of opposing reasons may partly reflect the disciplinary and ideological composition of the available literature. Therefore, this unexpected and surprisingly large difference in the number of opposing and supporting stances and reasons presented in the reviewed literature is not decisive for the ethical evaluation of GAS. This would require an investigation into the factual accuracy and the ethical weight of the reasons presented in the literature, which is beyond the objective of this review. Nevertheless, the critical tendency of the majority stances and reasons is remarkable considering the general acceptance of GAS by the medical/scientific community and by the public. At the same time, it is important to note here that we reviewed the normative ethical literature, and some of the reasons presented in the reviewed literature are at odds with scientific evidence. For instance, some reason-mentions formulate an ethical objection to GAS grounded in the assumption of widespread dissatisfaction and regret [24,26,32,38]. However, recent studies show that the regret rate after GAS is rather small compared to regret rates associated with some other common surgeries [76,77,78]. While presenting this as an example of a broader challenge in interpreting the reasons, the assessment of the empirical validity and scientific foundations of all reasons presented in this review falls beyond the scope of our study.

The various reasons for supporting or opposing GAS in relation to gender dysphoria analyzed in this review have shown that there are some overarching issues. Therefore, in addition to the general observations made above, we delve deeper into some specific underlying issues for further analysis and discussion.

### 4.1. Ambiguity in Understanding Gender Dysphoria

The analysis of various reasons presented in the first three categories, namely those related to the origin of gender dysphoria, treatment, and anthropology, shows that one of the issues underlying the debate is the different understandings and conceptualizations of gender dysphoria as an anthropological reality. Only some reason-mentions explicitly bring this anthropological understanding into the debate, in particular, those which endorse the unitive understanding against what they perceive as a dualistic anthropology [4,23,26,29,38,40,42]. Given that the majority of the literature reviewed here comes from theological and philosophical perspectives, the classical understanding of the human person in terms of the body–soul relationship can be seen as typical for these disciplines. This classical theological and philosophical understanding resonates in popular literature such as newspaper articles, where expressions such as “wrong body” and “mind-body disparity” have been frequently used [79,80,81,82]. At the same time, it should be noted that there are scholars who, from their anthropological perspective, reject such expressions as “wrong body” [83,84,85,86,87]. Given that there is no clear knowledge about the relationship between these two aspects, namely mind and body [25,40], divergent anthropologies remain a major cause of division among the authors from different disciplines.

### 4.2. Limited Exploration of the Origin of Gender Dysphoria

The mutually exclusive stances between the physical and psychological origins of gender dysphoria raise the question of which aspect, the body or the mind, should be corrected when there is a mismatch between them. The proposed solutions revolve around the choice between physical treatment such as surgery and psychological treatment such as psychotherapy. This suggests that ethical debates concerning GAS remain partly contingent upon an unresolved scientific question regarding the origin of gender dysphoria. At the same time, many studies in the medical literature in recent years have shed light on the multifactorial origin of gender dysphoria [88,89,90]. Thus, further exploration of the possibility of a multifactorial origin of gender dysphoria [39,44], including the mutually inclusive nature of physiological and psychological causes in the origin of gender dysphoria [4,49], provides new insights that might steer future ethical discussions in a different direction. At the same time, the opinion that the origin of gender dysphoria is not a good argument in the debate [4,39] also needs to be further elaborated.

### 4.3. Insufficient Integration of Different Perspectives on Sex and Gender

GAS as a solution to gender dysphoria implies certain conflicts with the traditional understandings of sex and gender. A critical question underlying GAS is whether sex can be changed and, if so, whether it should be changed [91]. The notions of sex and sex organs found in the reviewed literature led to the conclusion that being in the given sexed body is more important than the psychological satisfaction in a sexed body. Givenness as a norm also raises further questions about the autonomy and freedom of the individual to choose a psychologically satisfying life. Furthermore, though sex–gender dualism is referred to in some reason-mentions [35,38,47], the distinction between sex and gender and their nuanced understanding, which are widely discussed in modern transgender studies [92,93,94,95], seems to be insufficiently explored in the literature reviewed. This is symptomatic of the latter’s broader neglect of socio-cultural, political, phenomenological, legal, and more context-sensitive moral considerations. Greater conceptual clarity and engagement with contemporary interdisciplinary understandings may improve future ethical discussions regarding GAS.

### 4.4. Distress as a Focal Point

The experiential reality behind the phenomenon of gender dysphoria is the psychological suffering referred to as clinically significant distress [1]. The use of the term ‘strong distress’ in the *DSM-5-TR* emphasizes the need for an approach that is more oriented toward a practical solution that adequately addresses the suffering of those affected. Therefore, the effectiveness of the surgery in reducing distress is a primary concern when analyzing the ethical justification of GAS as a treatment option for gender dysphoria [33,39,91]. Although some reason-mentions suggest psychological methods, including psychotherapy, as a treatment option [27,41,42,44], the effectiveness of these methods in relieving the distress of individuals has also not been empirically proven. Further, while considering it supportive care, the major international diagnostic and clinical guideline documents [1,6,8] do not identify psychotherapy as a primary treatment option for gender dysphoria. Thus, on the one hand, the reasons analyzed in this review do not provide a definitive conclusion on the effectiveness of GAS in removing or reducing distress; on the other hand, they do not adequately address the distress as an experiential reality related to gender dysphoria. Future ethical analyses may benefit from examining more closely how the alleviation of distress should be weighed alongside other ethical considerations.

The limited focus on distress and its practical remedies is possibly related to the underemphasis on the principle of justice. Except for some financial concerns [37,41,43], the discussion of the principle of justice, which is one of the four important principles of the principlist approach [96], is missing from the whole discussion in the reviewed literature. Justice as an ethical principle demands giving each one that which is one’s due [43,96]. To ensure justice for people with gender dysphoria, it could be argued that their distress needs to be adequately addressed in ethical discussions.

Further, contemporary transgender studies show that distress may arise not only from individual experiences of incongruence but also from social experiences such as stigma, discrimination, lack of recognition, or barriers to social inclusion [97,98,99]. Thus, the integration of arguments based on the evidence-based literature and the inclusion of more transgender persons’ voices might shed more light on the distress and its appropriate remedy.

## 5. Conclusions

This systematic review identified and analyzed the ethical reasons both supporting and opposing GAS as a treatment option for gender dysphoria in adults. The review found a diverse and often conflicting body of normative ethical literature, encompassing five major categories of reasons. A substantial portion of the included literature was grounded in theological ethical perspectives, while comparatively few articles adopted non-theological ethical perspectives. After analyzing the ethical reasons in the available literature, we conclude that the current normative ethical literature is not yet sufficiently mature enough to reach a definite ethical conclusion regarding the ethical acceptability or unacceptability of GAS in adults. Thus, rather than establishing a definitive ethical conclusion, this final section outlines some unresolved questions and potential directions for future research emerging from this review. Some ambiguities identified in this review, including questions regarding the origin of gender dysphoria, the effectiveness of GAS, long-term outcomes, regret, and competing interpretations of beneficence and nonmaleficence, stem from normative assumptions that lack supporting empirical evidence. Excluding evidence from empirical literature, the majority of the authors rely on deductive reasoning, beginning with normative principles, which leads to abstract discussions rather than to concrete conclusions. Therefore, future ethical deliberations on this topic should include insights from various disciplines such as medicine, psychology, empirical ethics, and social sciences. By integrating more empirical findings about the lived experiences and opinions of the major stakeholders, a more comprehensive understanding of the ethical dilemmas surrounding GAS can be achieved, leading to a more informed and meaningful position in this complex discussion.

Taking the arguments of the multifactorial origin [39,44] and the mutually inclusive nature of the physiological and psychological origins [4,49] of gender dysphoria into consideration, it could be inferred that each case and every individual experience of gender dysphoria is unique. Given the multiple possible causes of gender dysphoria, including genetic, hormonal, developmental, psychological, social, and cultural factors and their combinations, the conclusion that GAS is a solution for all cases of gender dysphoria needs careful consideration. Although GAS could be a crucial component in the treatment of gender dysphoria, it may not be necessary or appropriate for every individual case. This idea aligns well with the World Professional Association for Transgender Health (WPATH) guidelines for GAS, which encourage an individualized approach to best meet a patient’s health care needs [8].

Similarly, while not negating the importance of respecting the individual’s autonomy and freedom to choose a psychologically satisfying life even in the absence of a strong distress, the argument that GAS is the last resort for an individual to have peace of mind [4,26] emerges as one of the important positions identified in the reviewed literature and warrants further ethical examination. The ethical nuances might vary significantly depending on whether GAS is adopted as the first treatment option for every case of gender dysphoria or whether it is accepted as a last resort for those to whom it is of vital importance. The treating physician could permit GAS as a last resort if other less invasive treatments are found to be ineffective in improving the patient’s psychological well-being [4]. Thus, the future ethical deliberations regarding GAS should depend on the careful assessment of each individual case of gender dysphoria.

## 6. Limitations

The literature search was restricted to papers that were peer-reviewed, published in English, and indexed in the selected electronic databases. This might have resulted in the omission of gray literature that is not included in these databases and the literature written in other languages. Some of the literature might exist in the form of books, manuscripts, published theses, dissertations, etc. In addition, as GAS is part of the wider gender-affirming health care practices, the literature on gender-affirming health care may contain some ethical discussions about gender transition that could be applied to gender-affirming surgery. As this article focuses specifically on GAS in adults, it did not include articles on children, adolescents, or young people, which may also contain ethical discussions. A substantial portion of the retrieved articles originated from the theological and philosophical ethical literature, which may not fully capture the diversity of contemporary ethical debates regarding GAS.

## Figures and Tables

**Table 1:** The details of the included articles.

Ref.	Author(s)	Title	Journal	Opinion of the Author(s)	Country of Affiliation of the First Author	Year of Publication	Perspective
[22]	Bayley, C.	“Transgender persons and Catholic healthcare”	*Health Care Ethics USA*	In favor of GAS	USA	2016	Theological
[23]	Bedford, E.L.; Eberl, J.T.	“Is the soul sexed? Anthropology, transgenderism, and disorders of sex development”	*Health Care Ethics USA*	Against GAS	USA	2016	Theological
[24]	Bizic, M.R.; Jeftovic, M.; Pusica, S.; Stojanovic, B.; Duisin, D.; Vujovic, S.; Rakic, V.; Djordjevic, M.L.	“Gender dysphoria: Bioethical aspects of medical treatment”	*Biomed Research International*	GAS on certain conditions	Serbia	2018	Philosophical
[25]	Brehany, J.F.	“Pope Pius XII and justifications for sex reassignment surgery”	*Health Care Ethics USA*	Against GAS	USA	2019	Theological
[26]	Brugger, E.C.	“Catholic hospitals and sex reassignment surgery: A reply to Bayley and Gremmels”	*The National Catholic Bioethics Quarterly*	Against GAS	Australia	2016	Theological
[27]	Bucar, E.M.	“Bodies at the margins: The case of transsexuality in Catholic and Shia ethics”	*Journal of Religious Ethics*	No definite position on GAS	USA	2010	Interreligious
[28]	Castaño, C.A.M.; Labrador, C.K.Q.; Piamonte, C.N.D.	“A Cynic and Epicurean take on the morality of sex change”	*Social Ethics Society Journal of Applied Philosophy*	Against GAS	Philippines	2018	Philosophical
[29]	Daly, T.T.W.	“Gender dysphoria and the ethics of transsexual (i.e, gender reassignment) surgery”	*Ethics & Medicine*	No definite position on GAS	USA	2016	Theological
[30]	Di Camillo, J.A.	“Gender transitioning and Catholic health care”	*The National Catholic Bioethics Quarterly*	Against GAS	USA	2017	Theological
[31]	Fitzgibbons, R.P.	“The desire for a sex change”	*Ethics & Medics*	Against GAS	USA	2009	Theological
[32]	Fitzgibbons, R.P.	“Transsexual attractions and sexual reassignment surgery: Risks and potential risks”	*The Linacre Quarterly*	Against GAS	USA	2005	Theological
[33]	Fitzgibbons, R.P.; Sutton, P.M.; O’Leary, D.	“The psychopathology of “sex reassignment” surgery: Assessing its medical, psychological, and ethical appropriateness”	*The National Catholic Bioethics Quarterly*	Against GAS	USA	2015	Theological
[34]	Furton, E.J.	“Catholic teaching on sex-reassignment: A reply to Carol Bayley”	*Ethics & Medics*	Against GAS	USA	2017	Theological
[35]	Furton, E.J.	“Confusion about sex and gender”	*Ethics & Medics*	Against GAS	USA	2016	Theological
[36]	Furton, E.J.	“A critique of “gender dysphoria” in *DSM-5*”	*Ethics & Medics*	Against GAS	USA	2015	Theological
[37]	Gremmels, B.	“Sex reassignment surgery and the Catholic moral tradition: Insight from Pope Pius XII on the principle of totality”	*Health Care Ethics USA*	In favor of GAS	USA	2016	Theological
[38]	Gross, C.	“Karol Wojtyla on sex reassignment surgery: An application of his philosophical anthropology”	*The National Catholic Bioethics Quarterly*	Against GAS	USA	2009	Philosophical
[39]	Guevin, B.M.	“Sex reassignment surgery for transsexuals: An ethical conundrum?”	*The National Catholic Bioethics Quarterly*	Against GAS	USA	2005	Philosophical
[40]	Harrison, J.	“Karol Wojtyla, sex reassignment surgery, and the body-soul union”	*The National Catholic Bioethics Quarterly*	Against GAS	USA	2017	Philosophical
[41]	Hume, M.C.	“Sex, lies, and surgery: The ethics of gender reassignment surgery”	*Res Cogitans*	GAS on certain conditions	USA	2011	Philosophical
[42]	Izunwa, M.O.	“Sex-reassignment surgeries and transsexual marriages: An ethical critique in the light of natural law”	*PREORC Journal of Arts and Humanities*	Against GAS	Nigeria	2018	Philosophical
[4]	Jones, D.A.	“Gender reassignment surgery: A Catholic bioethical analysis”	*Theological Studies*	No definite position on GAS	UK	2018	Theological
[43]	Latham, J.	“Ethical issues in considering transsexual surgeries as aesthetic plastic surgery”	*Aesthetic Plastic Surgery*	In favor of GAS	Australia	2013	Philosophical
[44]	Lindevaldsen, R.M.	“An ethically appropriate response to individuals with gender dysphoria”	*Liberty University Law Review*	Against GAS	USA	2019	Legal
[45]	McQueen, M.	“Catholic teaching on transgender (gender dysphoria)”	*Ethics Matters*	Against GAS	Canada	2016	Theological
[46]	Moschella, M.	“Sexual identity, gender, and human fulfillment: Analyzing the “middle way” between liberal and traditionalist approaches”	*Christian Bioethics*	Against GAS	USA	2019	Philosophical
[47]	Moschella, M.	“Trapped in the wrong body? Transgender identity claims, body-self dualism, and the false promise of gender reassignment therapy”	*The Journal of Medicine and Philosophy*	Against GAS	USA	2021	Philosophical
[48]	Stephens, T.R.	“The principle of totality does not justify sex reassignment surgery”	*Ethics & Medics*	Against GAS	USA	2016	Theological
[49]	Tonti-Filippini, N.	“Sex reassignment and Catholic schools”	*The National Catholic Bioethics Quarterly*	Against GAS	Australia	2012	Theological

## Data Availability

No new data were created or analyzed in this study. Data sharing is not applicable to this article.

## Supplementary Materials

The following supporting information can be downloaded at https://www.mdpi.com/article/10.3390/healthcare14162605/s1. Table S1: Codebook; Table S2: Concepts and related terms for literature search; Section S1: Search Strings Developed.

## Abbreviations

The following abbreviations are used in this manuscript:

*DSM-5-TR*	*Diagnostic and Statistical Manual of Mental Disorders, Fifth Edition, Text Revised*
*DSM-5*	*Diagnostic and Statistical Manual of Mental Disorders, Fifth Edition*
GAS	Gender-affirming Surgery
WPATH	World Professional Association for Transgender Health
