# Supplementary material for: Gender-Affirming Surgery as a Treatment Option for Gender Dysphoria in Adults: A Systematic Review of Ethical Reasons
Source: Healthcare (Basel). 2026 Aug 19;14(16):2605. doi: 10.3390/healthcare14162605 (PMC13512629; doi:10.3390/healthcare14162605)
Supplement: Supplementary file 1 [file healthcare-14-02605-s001.zip › healthcare-4417260-supplementary.pdf]

Supplementary Materials

GAS - Ethical Reasons

CODEBOOK

Table S1: Codebook

(The reasons supporting GAS are highlighted in green, whereas the reasons opposing GAS are highlighted in blue.)

No.	Code	Files	Reason-mentions
Origin and Nature of Gender Dysphoria			
Physiological			
1.	GD is a biological, physical or physiological disorder, because of the presence of sex organs	3	4
2.	Empirical evidences suggest physiological origin: Brain structures, variation is genes, sex-atypical brain	2	2
3.	Biological origin leads to biological determinism	2	2
Psychological			
4.	Psychological disorder, as biological origin is not established	7	10
5.	GD is related to mind's perception of the body rather than anything wrong with body	1	1
Multifactorial			
6.	Origin of GD is not a good argument in the debate	1	1
7.	Biological causes are minor, or only one part of the problem	2	2
8.	Biological and psychological factors are interrelated	1	1
9.	GD is multi factorial	2	2
10.	Social, developmental, environmental factors	3	3
Treatment for GD			
Physical			
11.	GAS alleviates the distress	2	2
12.	GAS is a corrective surgery, altering wrong organs	5	6
13.	GAS removes the issues with sex-atypical brain	1	1
14.	GAS is against marriage	5	7
15.	Sex change is not physiologically or medically possible	5	5
16.	Essential nature of the person cannot be changed, sex is more than organs	2	2
17.	GAS cannot lead to normality (sexual role, gametes), produces only a simulation or imitation of sexual organs	8	8
18.	GAS is against natural reproduction and sexual union	1	1
19.	No empirical evidence of sex-atypical brain	1	1
20.	Promotion of GAS prevent the development of other therapies	3	3
21.	Disorder driven demand, disordered perception of self, collaborating mental disorder	4	7
22.	Healthy genitalia is consider a disorder	1	1
Psychological			
23.	Psychotherapy over surgery	4	4
24.	GAS is not a real treatment	4	6
25.	Psychotherapy is in accordance with that sex is an immutable traits	1	1
26.	Psychotherapy avoids risks associated with surgery	1	1
27.	People with GD later become comfortable with their birth sex	1	1
28.	Psychotherapy follow the principle "first do no harm"	1	1
29.	Optimism of discovering appropriate psychological solution	1	1
Anthropological Reasons			

Understanding of the human person			
30.	Body-soul dualism is not possible or unity of body-soul	4	4
31.	Given body vs perceived self	1	1
32.	GAS involves body -self dualism	5	5
33.	GAS causes ontological disintegrity	2	2
34.	Sex-gender dualism is not possible	3	3
35.	GAS is an attempt to body-soul unity or is a story of integration	2	2
Givenness of the body			
36.	Rejection, devaluation, and dishonour the goodness of the given body	3	5
37.	Rejection of personhood and erroneous anthropology	2	2
38.	Givenness of gender	1	1
Effects of GAS			
Well-being of the individual			
39.	GAS alleviates the suffering causing by GD	3	3
40.	GAS leads to psychological stability and wellbeing	3	3
41.	GAS enhance self-esteem, self-image	1	1
42.	GAS is a futile treatment: unable to create functional organs	3	3
43.	No empirical data on benefits or long-term effects of GAS	10	10
44.	GAS does not remove the under lying issues	6	6
Life-saving			
45.	Suicide rate is high among GD people, GAS is life-saving	1	1
46.	GAS as a last resort when there is no other alternative	1	1
47.	Suicidal ideation is part of mental disorder	3	3
48.	Post surgery suicide rate is high	2	2
49.	Suicide threat as a blackmail	1	1
Falsehood and deception			
50.	GAS is based on a fantasy or involves falsehood	3	3
51.	GAS deceives oneself and others	3	3
52.	GAS satisfies only a fantasy desire	2	3
53.	GAS sometimes causes massive deception	1	1
54.	Feminist critique: Trans woman cannot claim to be a woman	1	1
55.	Reliability/face value of the claim	1	1
56.	Fantasy is part of the syndrome, GD	1	1
57.	GD is not a delusion as it is not a fixed idea	1	1
58.	Deception need not be always dishonest, individuals can be truthful with GAS	2	2
59.	Doctor can have truthful reason to permit surgery	2	2
Mutilation			
60.	Principle of totality to justify GAS	3	3
61.	GAS involves mutilation or destruction of healthy sexual organs	9	9
62.	Principle of totality cannot be applied in the case of GAS	11	11
Sterility			
63.	GAS leads to sterilization and infertility	3	3
64.	GAS leads to artificial reproduction	1	1
65.	Principle of double effect cannot be applied in GAS	4	4
66.	More than simple sterilization	1	1
67.	GAS is not direct (directed to) sterilization	1	1
68.	Principle of double effect in the case resulting sterility in GAS	1	1
Regret and Dissatisfaction			
69.	Individuals are dissatisfied and regret after surgery, demand for reversal	4	4
70.	Rate of regret is very low or occasional	3	3
71.	Regret is not because of surgery result, but because of social acceptance	1	1
Ethical Principles			
Autonomy			
72.	GAS is according to the autonomy of the patient	4	4
73.	Surgery is permissible under certain conditions	1	1

74.	Autonomy is questioned in case of mental disorder	1	1
75.	GAS is based on a distorted freedom	2	2
76.	Hope, desires and expectations may not correlate with the reality	1	1
77.	GAS denies the volitional power of the will	1	1
78.	Autonomy because of need based analysis	1	1
Nonmaleficence			
79.	Violation of the principle First do no harm	6	6
80.	Surgery does more harm than good	3	3
81.	Comorbidities involved	3	3
82.	Evidence that long-term effects are deleterious	1	1
83.	Surgery does not address real issues, causes further psychological issues	1	1
84.	Clinical conditions admit exceptions	1	1

Table S2. Concepts and related terms for literature search.

Group A: Gender-Affirming				
No.	Concepts	MeSH Terms	Term	Grouping
1.	Sex affirmation/sex affirming	No relevant MeSH	(sex* AND affirm*)	((sex OR gender) AND (affirm* OR confirm* OR assign* OR reassign* OR chang* OR transform*))
2.	Sex confirmation/sex confirming	"Sex Reassignment Surgery"[Mesh]	(sex* AND confirm*)	
3.	Sex assignment	No relevant MeSH	(sex* AND assign*)	
4.	Sex reassignment	"Sex Reassignment Surgery"[Mesh] "Sex Reassignment Procedures"[Mesh]	(sex* AND reassign*)	
5.	Sex change	"Sex Reassignment Surgery"[Mesh]	(sex* AND chang*)	
6.	Sex transformation	No relevant MeSH	(sex* AND transform*)	
7.	Gender affirmation	"Sex Reassignment Surgery"[Mesh] "Sex Reassignment Procedures"[Mesh]	(gender AND affirm*)	
8.	Gender confirmation/gender confirming	"Sex Reassignment Surgery"[Mesh] "Sex Reassignment Procedures"[Mesh]	(gender AND confirm*)	
9.	Gender reassignment	"Sex Reassignment Surgery"[Mesh] "Sex Reassignment Procedures"[Mesh]	(gender AND reassign*)	
10.	Gender transformation	No relevant MeSH	(gender AND transform*)	
11.	Gender change	"Sex Reassignment Surgery"[Mesh] "Sex Reassignment Procedures"[Mesh]	(gender AND chang*)	
12.	Genital reassignment	No relevant MeSH	(genital* AND reassign*)	
13.	Genital reconstruction	No relevant MeSH	(genital* AND reconstruct*)	
14.	Genital	"Genitalia"[Mesh]	genital*	
15.	Bottom	No relevant MeSH	bottom	
16.	Top	No relevant MeSH	top	

17.	Facial/neck/chest femini(z)ation	"Feminization"[Mesh]	feminiz*<br>feminis*
18.	Facial/neck/chest masculinization	No relevant MeSH	masculiniz*<br>masculinis*
19.	Male-to-female	No relevant MeSH	male-to-female
20.	Male to female	No relevant MeSH	"male to female"
21.	MtF	No relevant MeSH	mtf
22.	Female-to-male	No relevant MeSH	female-to-male
23.	Female to male	No relevant MeSH	"female to male"
24.	FtM	No relevant MeSH	ftm
25.	Transsexual	"Transgender Persons"[Mesh]<br>"Transsexualism"[Mesh]	transsexual*
26.	Transgender	"Transgender Persons"[Mesh]<br>"Transsexualism"[Mesh]	transgender*
27.	Feminizing/Masculinizing genitoplasty	No relevant MeSH	genitoplast*
Group B: Surgery			
No	Concepts	MeSH	Term
1	Surgery/surgeries/surgical	"General Surgery"[Mesh]<br>"Surgical Procedures, Operative"[Mesh]	surger*<br>surgic*
2.	Operation/operations	"Surgical Procedures, Operative"[Mesh]	operat*
Group C: Ethics			
No	Concepts	MeSH	Term
1.	Ethic/ethics/ethical	"Ethics"[MeSH]<br>"Ethics, Clinical"[Mesh]	ethic*
2.	Moral/morality	"Morals"[MeSH]	moral*
3.	Bio-ethic/bio-ethics/bio-ethical	No relevant MeSH	bio-ethic*
4.	Bioethics/bioethics/bioethical	"Bioethics"[MeSH]	bioethic*
5.	Medical ethic/medical ethics/medical ethical	"Ethics, Medical"[MeSH]	"medical ethic*"
6.	Biomedical ethic/biomedical ethics/biomedical ethical	"Bioethics"[MeSH]	"biomedical ethic*"

Section S1. Search Strings Developed

PubMed

((("Sex Reassignment Surgery"[Mesh] OR "Sex Reassignment Procedures"[Mesh]) OR (("Genitalia"[Mesh] OR "Feminization"[Mesh] OR "Transgender Persons"[Mesh] OR "Transsexualism"[Mesh] OR ((sex OR gender) AND (affirm* OR confirm* OR assign* OR reassign* OR chang* OR transform*)) OR genital*[tiab] OR bottom[tiab] OR top[tiab] OR feminiz*[tiab] OR feminis*[tiab] OR masculiniz*[tiab] OR masculinis*[tiab] OR male-to-female[tiab] OR mtf[tiab] OR "male to female"[tiab] OR female-to-male[tiab] OR "female to male"[tiab] OR ftm[tiab] OR transsexual*[tiab] OR transgender*[tiab] OR genitoplast*[tiab]) AND ("General Surgery"[Mesh] OR "Surgical Procedures, Operative"[Mesh] OR surger*[tiab] OR surgic*[tiab] OR

operat*[tiab]))) NOT (cancer*[tiab] OR cosmetic*[tiab] OR minor*[tiab] OR child*[tiab] OR adolescen*[tiab])) AND ("Ethics"[MeSH] OR "Ethics, Clinical"[Mesh] OR "Morals"[MeSH] OR "Bioethics"[MeSH] OR "Ethics, Medical"[MeSH] OR ethic*[tiab] OR moral*[tiab] OR bio-ethic*[tiab] OR bioethic*[tiab] OR "medical ethic*" OR "biomedical ethic*") Filters: English, Humans, from 2002/1/1–2022/12/31

Embase

#1—'gender affirming surgery'/exp OR 'facial feminization surgery'/exp OR 'feminization surgery'/exp OR 'masculinization surgery'/exp OR 'feminizing genitoplasty'/exp OR 'masculinizing genitoplasty'/exp OR genitoplast*:ab,ti
#2—'gender reassignment'/exp OR 'gender affirmation'/exp OR 'gender transition'/exp OR 'genital reconstruction'/exp OR 'transgender'/exp OR ((sex*:ab,ti OR gender:ab,ti) AND (affirm*:ab,ti OR confirm*:ab,ti OR assign*:ab,ti OR reassign*:ab,ti OR chang*:ab,ti OR transform*:ab,ti)) OR (genital*:ab,ti AND reassign*:ab,ti) OR (genital*:ab,ti AND reconstruct*:ab,ti) OR genital*:ab,ti OR bottom:ab,ti OR top:ab,ti OR feminiz*:ab,ti OR feminis*:ab,ti OR masculiniz*:ab,ti OR masculinis*:ab,ti OR male-to-female:ab,ti OR 'male to female':ab,ti OR mtf:ab,ti OR female-to-male:ab,ti OR 'female to male':ab,ti OR ftm:ab,ti OR transsexual*:ab,ti OR transgender*:ab,ti
#3—'surgery'/exp OR surger*:ab,ti OR surgic*:ab,ti OR operat*:ab,ti
#4—cancer*:ab,ti OR cosmetic*:ab,ti OR minor*:ab,ti OR child*:ab,ti OR adolescen*:ab,ti
#5—'ethics'/exp OR 'morality'/exp OR 'bioethics'/exp OR 'medical ethics'/exp OR ethic*:ab,ti OR moral*:ab,ti OR bio-ethic*:ab,ti OR bioethics*:ab,ti OR 'medical ethic*':ab,ti OR 'biomedical ethic*':ab,ti
#6—#2 AND #3
#7—#1 OR #6
#8—#7 NOT #4
#9—#8 AND #5
#10—#8 AND #5 AND [english]/lim AND [2002–2022]/py

Scopus

TITLE-ABS-KEY ((((((sex* OR gender*) AND (affirm* OR confirm* OR assign* OR reassign* OR chang* OR transform* OR reconstruct*)) OR genital* OR bottom OR top OR feminiz* OR feminis* OR masculiniz* OR masculinis* OR male-to-female OR "male to female" OR mtf OR female-to-male OR "female to male" OR ftm OR transsexual* OR transgender*) AND (surger* OR surgic* OR operat*)) AND NOT (cancer* OR cosmetic* OR minor OR child* OR adolescen*)) AND (ethic* OR moral* OR bio-ethic* OR bioethic* OR "biomedical ethic*")) AND PUBYEAR > 2001 AND PUBYEAR < 2023 AND (LIMIT-TO (LANGUAGE, "English"))

ATLA Religion

(((((sex* OR gender) AND (affirm* OR confirm* OR assign* OR reassign* OR chang* OR transform* OR reconstruct*)) OR genital* OR bottom OR top OR feminiz* OR feminis* OR masculiniz* OR masculinis* OR male-to-female OR "male to female" OR mtf OR female-to-male OR "female to male" OR ftm OR transsexual* OR transgender*) AND (surger* OR

surgic* OR operat*)) NOT (cancer* OR cosmetic* OR minor OR child* OR adolescen*)) AND (ethic* OR moral* OR bio-ethic* OR bioethic*)

Index Theologicus

(((((sex* OR gender) AND (affirm* OR confirm* OR assign* OR reassign* OR chang* OR transform* OR reconstruct*)) OR genital* OR bottom OR top OR feminiz* OR feminis* OR masculiniz* OR masculinis* OR male-to-female OR "male to female" OR mtf OR female-to-male OR "female to male" OR ftm OR transsexual* OR transgender*) AND (surger* OR surgic* OR operat*)) NOT (cancer* OR cosmetic* OR minor OR child* OR adolescen*)) AND (ethic* OR moral* OR bio-ethic* OR bioethic*)

Philosopher's Index

(((((sex* OR gender) AND (affirm* OR confirm* OR assign* OR reassign* OR chang* OR transform* OR reconstruct*)) OR genital* OR bottom OR top OR feminiz* OR feminis* OR masculiniz* OR masculinis* OR male-to-female OR "male to female" OR mtf OR female-to-male OR "female to male" OR ftm OR transsexual* OR transgender*) AND (surger* OR surgic* OR operat*)) NOT (cancer* OR cosmetic* OR minor OR child* OR adolescen*)) AND (ethic* OR moral* OR bio-ethic* OR bioethic*)

Web of Science

((((ALL = ((sex* OR gender*) AND (affirm* OR confirm* OR assign* OR reassign* OR chang* OR transform* OR reconstruct*))) OR ALL = (genital* OR bottom OR top OR feminiz* OR feminis* OR masculiniz* OR masculinis* OR male-to-female OR "male to female" OR mtf OR female-to-male OR "female to male" OR ftm OR transsexual* OR transgender*)) AND ALL = (surger* OR surgic* OR operat*)) NOT ALL = (cancer* OR cosmetic* OR minor OR child* OR adolescen*)) AND ALL = (ethic* OR moral* OR bio-ethic* OR bioethic*) and English (Languages) and 2022 or 2021 or 2020 or 2019 or 2018 or 2016 or 2017 or 2015 or 2014 or 2013 or 2012 or 2011 or 2010 or 2009 or 2008 or 2007 or 2006 or 2005 or 2004 or 2003 or 2002 (Publication Years)
